# Dynamics of cattle sperm sncRNAs during maturation, from testis to ejaculated sperm

**DOI:** 10.1186/s13072-021-00397-5

**Published:** 2021-05-24

**Authors:** Eli Sellem, Sylvain Marthey, Andrea Rau, Luc Jouneau, Aurelie Bonnet, Chrystelle Le Danvic, Benoît Guyonnet, Hélène Kiefer, Hélène Jammes, Laurent Schibler

**Affiliations:** 1 R&D Department, ALLICE, 149 rue de Bercy, 75012 Paris, France; 2 AgroParisTech, INRAE, GABI, Université Paris-Saclay, 78350 Jouy-en-Josas, France; 3 UVSQ, INRAE, BREED, Université Paris Saclay, 78350 Jouy en Josas, France; 4 Ecole Nationale Vétérinaire D’Alfort, BREED, 94700 Maisons-Alfort, France; 5 R&D Department, Union Evolution, rue Eric Tabarly, 35538 Noyal-Sur-Vilaine, France; 6 BioEcoAgro Joint Research Unit, INRAE, Université de Liège, Université de Lille, Université de Picardie Jules Verne, Estrées-Mons, France; 7 INRAE, MaIAGE, Université Paris-Saclay, 78350 Jouy-en-Josas, France

**Keywords:** Sperm epigenetics, SncRNAs, Bovine, MiRNA, PiRNA, TRFs, RRFs, Epididymis location, Testicular parenchyma sperm

## Abstract

**Background:**

During epididymal transit, spermatozoa go through several functional maturation steps, resulting from interactions with epididymal secretomes specific to each region. In particular, the sperm membrane is under constant remodeling, with sequential attachment and shedding of various molecules provided by the epididymal lumen fluid and epididymosomes, which also deliver sncRNA cargo to sperm. As a result, the payload of sperm sncRNAs changes during the transit from the epididymis caput to the cauda. This work was designed to study the dynamics of cattle sperm sncRNAs from spermatogenesis to final maturation.

**Results:**

Comprehensive catalogues of sperm sncRNAs were obtained from testicular parenchyma, epididymal caput, corpus and cauda, as well as ejaculated semen from three Holstein bulls. The primary cattle sncRNA sperm content is markedly remodeled as sperm mature along the epididymis. Expression of piRNAs, which are abundant in testis parenchyma, decreases dramatically at epididymis. Conversely, sperm progressively acquires miRNAs, rsRNAs, and tsRNAs along epididymis, with regional specificities. For instance, miRNAs and tsRNAs are enriched in epididymis cauda and ejaculated sperm, while rsRNA expression peaks at epididymis corpus. In addition, epididymis corpus contains mainly 20 nt long piRNAs, instead of 30 nt in all other locations. Beyond the bulk differences in abundance of sncRNAs classes, K-means clustering was performed to study their spatiotemporal expression profile, highlighting differences in specific sncRNAs and providing insights into their putative biological role at each maturation stage. For instance, Gene Ontology analyses using miRNA targets highlighted enriched processes such as cell cycle regulation, response to stress and ubiquitination processes in testicular parenchyma, protein metabolism in epididymal sperm, and embryonic morphogenesis in ejaculated sperm.

**Conclusions:**

Our findings confirm that the sperm sncRNAome does not simply reflect a legacy of spermatogenesis. Instead, sperm sncRNA expression shows a remarkable level of plasticity resulting probably from the combination of multiple factors such as loss of the cytoplasmic droplet, interaction with epididymosomes, and more surprisingly, the putative in situ production and/or modification of sncRNAs by sperm. Given the suggested role of sncRNA in epigenetic trans-generational inheritance, our detailed spatiotemporal analysis may pave the way for a study of sperm sncRNAs role in embryo development.

**Supplementary Information:**

The online version contains supplementary material available at 10.1186/s13072-021-00397-5.

## Introduction

Spermatozoa are produced from germ cells in the seminiferous tubules of the male testes by a complex stepwise process called spermatogenesis. Spermatogenesis is a tightly regulated developmental process involving thousands of genes and proteins [1] as well as small non-coding RNAs (sncRNAs), especially micro-RNAs (miRNAs) and piwi-interacting RNAs (piRNAs) [2]. For instance, the selective knock-out of Dicer1 at the onset of male germ cell development leads to infertility, due to multiple cumulative defects at the meiotic and post-meiotic stages of spermatogenesis [3], highlighting the role of micro-RNAs (miRNAs) and endogenous small interfering RNAs (endo-siRNAs). Likewise, miR-100, the miR-29 family, and miR-34c have been shown to be required for spermatogonial stem cell proliferation [4], regulation of meiosis [5], and germinal expression profiles [6], respectively. Likewise, piRNAs are known to play roles in spermatogenesis, as evidenced by the mitosis, meiosis, chromatin compaction, flagella elongation, and fertility defects in mutants lacking Piwi [7].

Beyond spermatogenesis, spermatozoa go through several temporal functional maturation steps at various locations to acquire their fertilizing capacity. In particular, sperm gain motility and acrosomal function while in the epididymis. During epididymal transit, the sperm membrane is under constant remodeling, with sequential attachment and shedding of various molecules provided by the epididymal lumen fluid [8] and extracellular vesicles, as epididymosomes, including proteins [9, 10] and lipids [11]. In addition, sncRNA cargo are also delivered to sperm by epididymosomes [12, 13]. The head (caput), body (corpus), and tail (cauda) make up the three main regions of the epididymis, which can be distinguished by specific epithelial morphology, luminal diameter, protein [14, 15], and lipid content [11, 16], as well as expression pattern and epididymosome content [17–20]. In addition, miRNA repertoires contained within epididymosomes were shown to differ from those of their parent epithelial cells, suggesting an active sorting of miRNAs released into the intraluminal fluid [21].

Several biological roles have been proposed for epididymosomes, including intercellular communication throughout the epididymis [21], modulation of sperm motility during epididymal transit, protection against oxidative stress, acquisition by spermatozoa of surface proteins essential for fertilization, tagging defective sperm for elimination [22], and supporting embryo development [12, 19, 23]. Epididymal sperm maturation is thus the result of complex and sequential interactions with different epididymal secretomes specific to each region. As a result, the sperm proteome [24], lipid composition [25], and sncRNA payload [18, 19] change during the transit from the epididymis caput to the cauda.

Given the suggested role of sncRNA in the phenomenon of epigenetic trans-generational inheritance [26], this work was designed to study the dynamics of cattle sperm sncRNAs from spermatogenesis to final maturation. Our previous study provided a comprehensive overview of cattle sperm sncRNA classes including rRNA-derived small RNAs (rsRNAs), piRNAs, miRNAs, and tRNA-derived small RNAs (tsRNAs), and revealed an impressive diversity of isoforms (isomiRs, isopiRs) produced by RNA editing [27]. Here, comprehensive catalogues of sperm sncRNAs were obtained from gametes isolated from testicular parenchyma, epididymal caput, corpus, and cauda to infer the potential origin of sncRNAs observed in ejaculated sperm.

## Results

### Total RNA preparation and UMI NGS sequencing

At least 20 million spermatozoa could be recovered from each sampling region. Contamination with somatic cells was negligible, as confirmed by microscopy (less than 1 somatic cell per 1000 sperm cells) and the *GPX5* ddCts obtained from a comparison of RNA expression between sperm and somatic cells (control sample).

About 23 to 1970 ng of total RNA could be obtained from sperm, depending on the sampling region. Consistent amplifications of bta-miR-125 (Ct in the range 24–26 starting from 5 ng of total RNA) and single peak melt curves were obtained.

Sequencing resulted in 388,357,674 raw sequence reads, with on average 32.6 ± 7.7 million reads per library. After de-duplication based on UMIs and filtering, 199,642,474 reads were kept, with on average 13.3 ± 9.1 million reads per library, corresponding to 1,088,318 unique reads. On average, about 80% of sequences could be mapped unambiguously to the cattle reference genome, while 20% were outmapped (more than five genomic locations, e.g., mapped to repetitive sequences) or unmapped (Additional file 3: Table S1).

As described in “Methods” section, reads were annotated using several databases and classified as micro-RNAs (miRNAs), Piwi-interacting RNAs (piRNAs or piRNA-like), tsRNAs, rsRNAs, circular RNAs (circRNAs), mRNAs, and other RNAs. Sperm sncRNA comprises a wide variety of sncRNA classes, mainly miRNAs, piRNAs, tsRNAs, and rsRNAs (Additional file 3: Tables S2-S5). Reads annotated as mRNAs accounted for only 0.8% (ranging from 3000 to 17,000 reads, according to the sampling region), highlighting the absence of somatic cell RNA contaminations. Only a small proportion of reads (5.6%) remained unannotated.

### The sncRNA signature discriminates between sperm sampled along the male tract

**Fig. 1:**
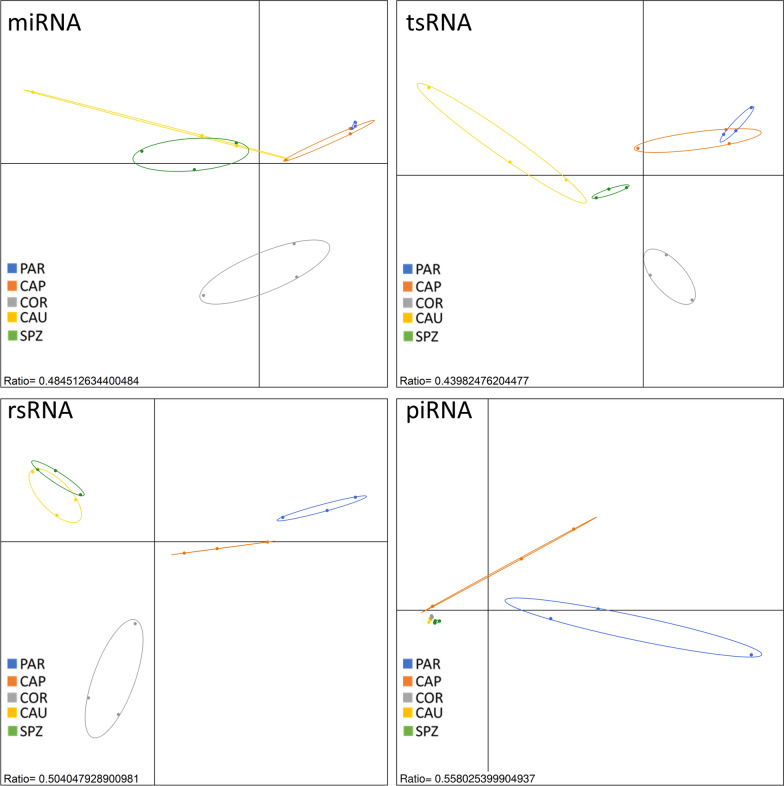
sncRNA signature discriminates between sperm sampled along the male tract. Regions could be distinguished by Between Class Analysis (BCA), regardless of the sncRNA family, except for piRNA which failed to discriminate between epididymis corpus (COR), cauda (CAU), and ejaculated sperm (SPZ). Interestingly, testis parenchyma (PAR) and epididymis caput (CAP) on one hand as well as epididymis cauda and ejaculated sperm on the other hand, appeared to be close together, while epididymis corpus exhibited a distinct profile

A between-class analysis (BCA, Fig. 1) showed considerable differences in sperm sncRNA content according to the sampling region, making it possible to discriminate between sperm samples collected at various developmental stages along the male tract. As exemplified by between-class inertia percentages (ratio ranging from 44 to 56%) within each sncRNA family, a large proportion of variance could be attributed to the sampling region. Interestingly, testis parenchyma (PAR) and epididymis caput (CAP) on one hand as well as epididymis cauda (CAU) and ejaculated sperm (SPZ) on the other hand, appeared to be close together regardless of the sncRNA family. Epididymis corpus (COR) showed peculiarities in terms of miRNA, tsRNA, and rsRNA content, and were clearly distinct from the two other groups. No differences could be highlighted based on piRNA content, except for PAR and CAP. These observations strongly suggest that sperm sncRNA content is highly dynamic, with sncRNA families exhibiting particular trends along the male tract.

### Overall dynamics of cattle sperm sncRNAs from spermatogenesis to final maturation

**Fig. 2:**
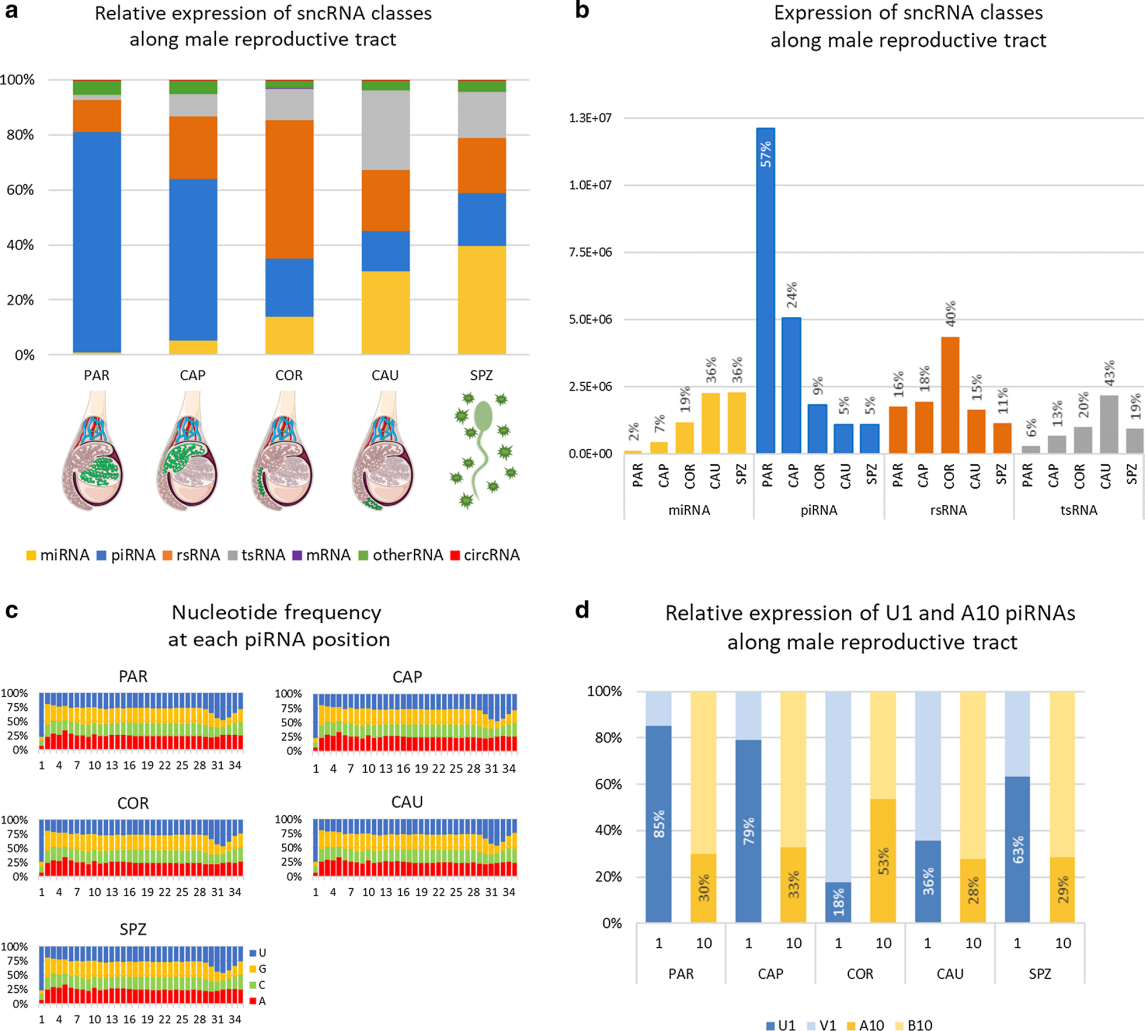
Changes in sncRNA expression along the male reproductive tract. **a** Relative expression of each sncRNA class was computed for each of the five regions as the proportion (%) of the class expression relative to the total expression in the region: PAR (testis parenchyma), CAP (caput), COR (corpus), and CAU (cauda) epididymis and ejaculated sperm (SPZ). The bar graph clearly illustrates opposing trends between piRNAs, whose proportion decreases from parenchyma (78%) to cauda (13%) and ejaculated sperm (18%), and miRNAs and tsRNA, which follow an overall upward trend from a few % in parenchyma to 36% and 16% in ejaculated sperm, respectively. **b** Normalized expression level of each sncRNA class along the male tract. The proportion of total expression in each sampling regions is indicated above each bar for each sncRNA class (e.g., PAR and SPZ accounts for 2% and 36% of miRNA expression, respectively). **c** Nucleotide frequency at each position for all detected piRNA in each region. For each region, the frequency of each nucleotide was computed along the piRNA sequence and plotted as bar charts (from the 1st nucleotide at 5ʹ end to the 35th nucleotide). More than 75% of piRNA were shown to possess a U residue at their 5ʹ end (U1), and no enrichment for an A residue at position 10 (A10) was observed. **d** Relative expression of U1 relative to non-U1 piRNAs (V1) as well as A10 relative to non-A10 (B10) piRNA was computed per region. U1 piRNAs generated via the primary production pathway account for the majority of piRNA expression except at COR and CAU where piRNA expression is dominated by non-U1 piRNAs. Likewise, A10 piRNAs account for about 25% of piRNAs, except at COR where A10 piRNAs account to 60% of expression. B: IUPAC degenerate base symbols for not A, and V for not U

As illustrated by Fig. 2a, b, sperm sncRNA content varies greatly between the different regions. In particular, piRNAs and piRNA-like were shown to account for 78% of all sncRNAs at PAR and decrease along the epididymis from 57% at CAP to 13% at CAU. A slight increase in proportion was observed in ejaculated sperm (18%).

The nucleotide composition of the piRNA pool was assessed for each of the five sampling regions to gain insight into the mechanism through which piRNAs were generated during sperm transit. Notably, regardless of the region, piRNAs were determined to bear predominantly a uracil (U) residue at their 5ʹ end (U1 piRNAs), and no enrichment for an A residue at piRNA position 10 (A10) was observed (Fig. 2c). Of note, if U1 piRNAs account for a vast majority of piRNAs for all regions, considerable variation in their relative expression was observed across regions (Fig. 2d and Additional file 3: Table S6). Indeed, while they account for about 80% of the expression of piRNAs in PAR and CAP, this ratio was almost inverted in COR and the expression of piRNAs was shown to be dominated by non-U1 piRNAs in COR and CAU. Likewise, A10 enrichment was observed at COR where A10 piRNAs account for about 60% of expression. Taken together, these findings suggest that sperm piRNAs are acquired by different mechanisms along the male tract.

**Fig. 3:**
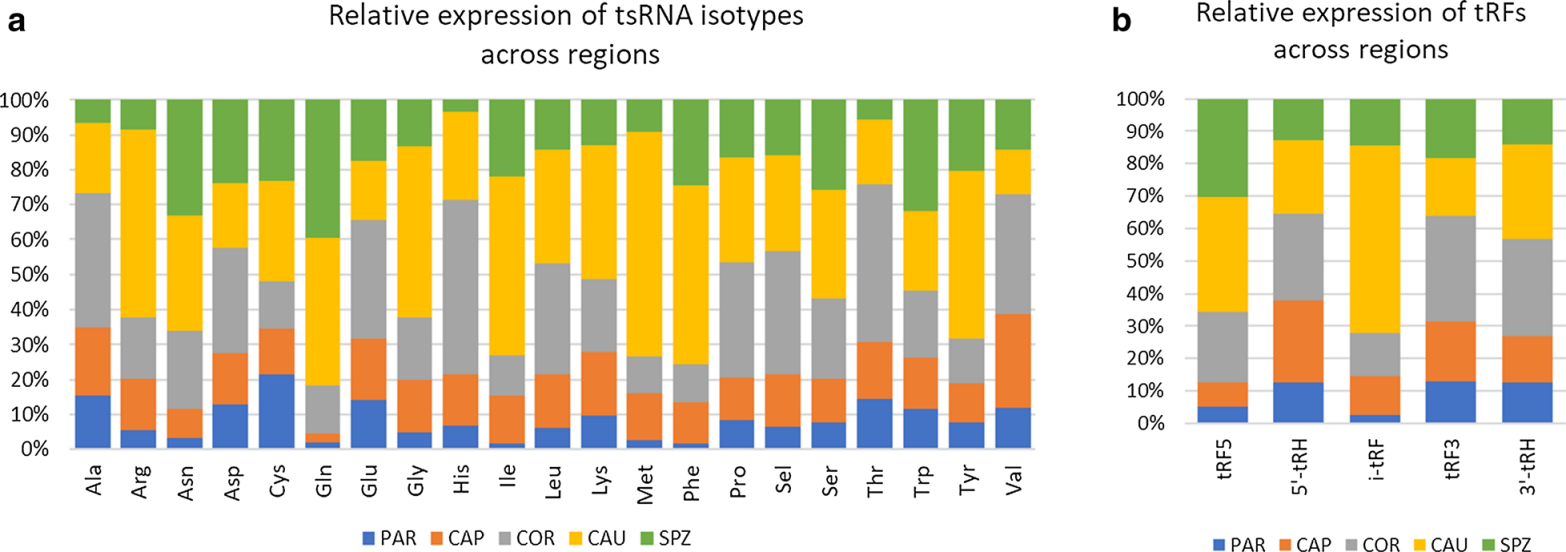
Dynamics of tsRNAs across regions. PAR (testis parenchyma), CAP (caput), COR (corpus), CAU (cauda) epididymis, and ejaculated sperm (SPZ). **a** Distribution across regions of isoacceptor read counts, showing specific expression patterns. For instance, Alanine, Histidine, and Threonine are mainly expressed in COR, while Arginine, Glycine, Isoleucine, and Methionine are mostly expressed in CAU. **b** Distribution across regions of tRNA-derived fragments showing that tRF5s are mainly expressed in CAU (35%) and SPZ (30%), while i-tRFs are mainly expressed in CAU (58%). tRF5 and tRF3: fragments aligned to the 5′ or 3’ end of the mature tRNA; 5′-tRHs and 3′-tRHs: 5’ or 3’ tRNA halves generated by a cleavage at the anticodon-loop; i-tRFs: internal fragments

In contrast to piRNAs, miRNA content increased along the male tract. Indeed, while miRNA account for only 1% of sncRNA expression at PAR, increasing miRNA expression was observed along the epididymis (5%, 13%, and 27% in CAP, COR, and CAU, respectively) to reach 38% in ejaculated sperm. Likewise, tsRNA content increased from PAR (2% of sncRNA expression) to CAU (26%). Isoforms associated with 21 amino acids were identified, but four isoacceptors contributed to 69% of all identified tsRNAs (Additional file 3: Table S7): glycine (29% of read counts), Methionine (18%), Glutamine (14%), and Serine (10%). Differences in their relative expression were observed across regions, as illustrated in Fig. 3a. For instance, Alanine, Histidine and Threonine are mainly expressed at COR, while Arginine, Glycine, Isoleucine, and Methionine are mostly expressed at CAU. Glutamine and Glutamate isoacceptors account for 30% of expressed tsRNA in SPZ, while they account for only 8% in CAP. Glycine isoacceptor is the most expressed tsRNA regardless of the region, ranging from 20% in SPZ to 35% in CAU. Likewise, tsRNA sub-groups showed particular expression profiles (Fig. 3b), as exemplified by tRF5s, which are mainly expressed at CAU (35%) and SPZ (30%), while i-tRFs are mainly expressed in CAU (58%). Altogether, i-tRFs accounted for 46% of tRFs expression, ranging from 19% at PAR to 62% at CAU.

**Fig. 4:**
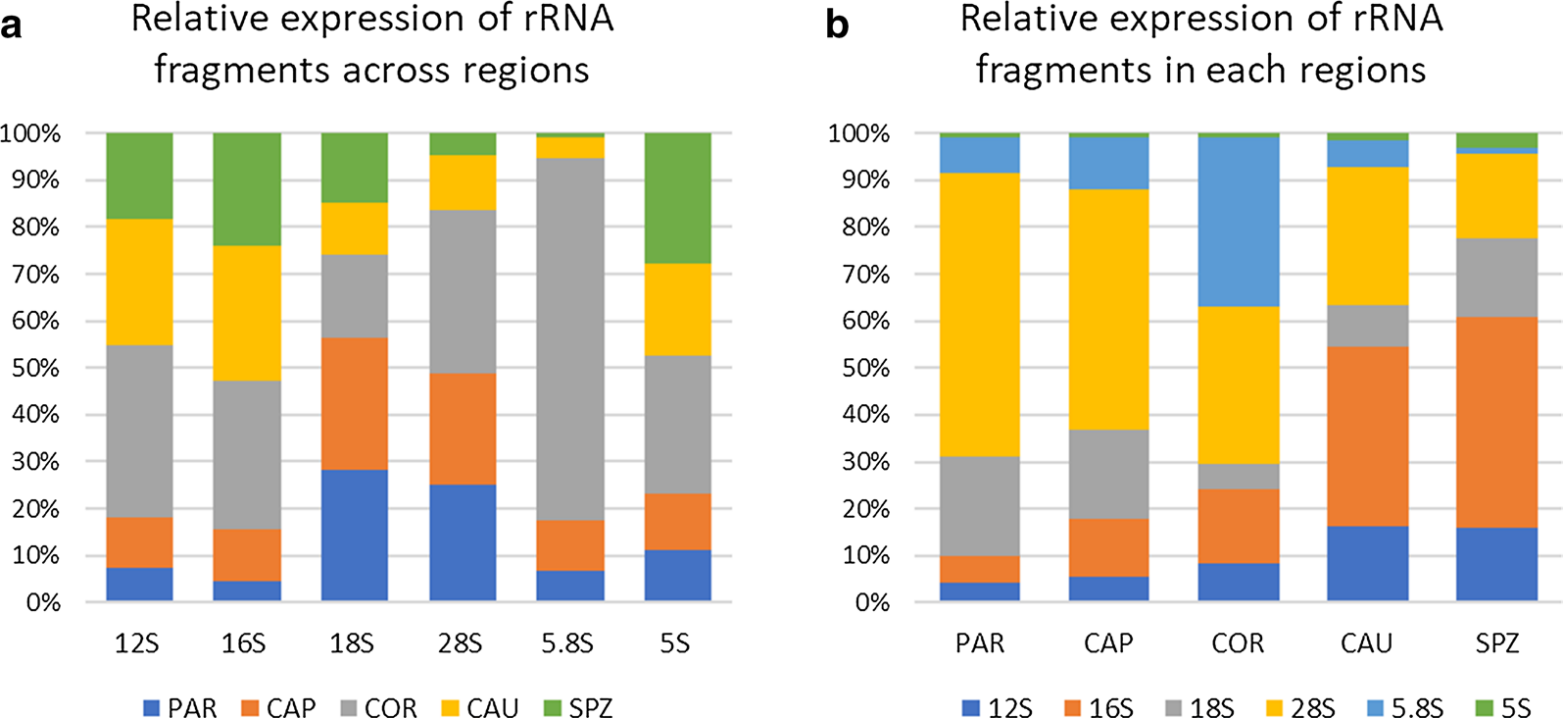
Dynamics of rRNA fragments across regions. PAR (testis parenchyma), CAP (caput), COR (corpus), and CAU (cauda) epididymis and ejaculated sperm (SPZ). **a** Relative expression of reads counts from each region was computed per rRNA fragment subtype, illustrating specific patterns of rRNA expression. For instance, 5.8S derived fragments are primarily expressed in epididymis corpus. **b** Relative expressions of rRNA fragment subtypes were computed per region, showing that fragments derived from 28s rRNA account for the majority of read counts in parenchyma and epididymis caput, while fragments derived from 16s rRNA account for a large proportion of read counts in epididymis cauda and ejaculated sperm

While piRNA, miRNA, and tRNA expression seemed to follow an overall downward or upward trend, rRNAs showed a particular profile, peaking at COR (46% of sncRNA expression). Specific expression patterns were observed, as illustrated in Fig. 4 and Additional file 3: Table S8: fragments derived from 28s rRNA account for the majority of expression in PAR (60%) and CAP (51%), while fragments derived from 16s rRNA account for a large proportion of read counts in CAU (38%) and SPZ (45%). Of note, while 5.8S represent only 2% of the different rsRNA species at COR, they account for 36% of expression in this region. Other sncRNAs did not display great differences among regions, accounting for only a small proportion of sperm sncRNAs.

### In depth study of sperm sncRNA differentially expressed along male reproductive tract

A differential expression analysis was performed using DESeq2 for all pairwise comparisons between sampling regions. Table 1 summarizes the number of differentially expressed sncRNA between each region.

**Table 1:** Number of sperm sncRNAs differentially expressed (adjusted *p* values < 0.05, fold difference > ± 2.5) between the five sampling regions: testis parenchyma (PAR), epididymis caput (CAP), corpus (COR), cauda (CAU), and ejaculated sperm (SPZ)

Testis	Epididymis			Ejaculated sperm
PAR	CAP	COR	CAU	SPZ
PAR	4436	156,611	120,914	194,062
	CAP	52,471	77,264	82,921
		COR	5209	21,074
			CAU	1188
				SPZ

Consistent with the aforementioned global trends, the number of differentially expressed sncRNA was shown to increase with the spatiotemporal distance between samples: while only 1188 differentially expressed sncRNAs were observed between SPZ and CAU, comparisons of SPZ with sperm sampled at COR, CAP, and PAR highlighted 21,074, 82,921, and 194,062 differentially expressed sncRNAs, respectively. Over-expression in SPZ compared to PAR of five isomiRs was confirmed by RT-qPCR (Additional file 3: Table S9), therefore validating the parameters used for differential analysis.

**Fig. 5:**
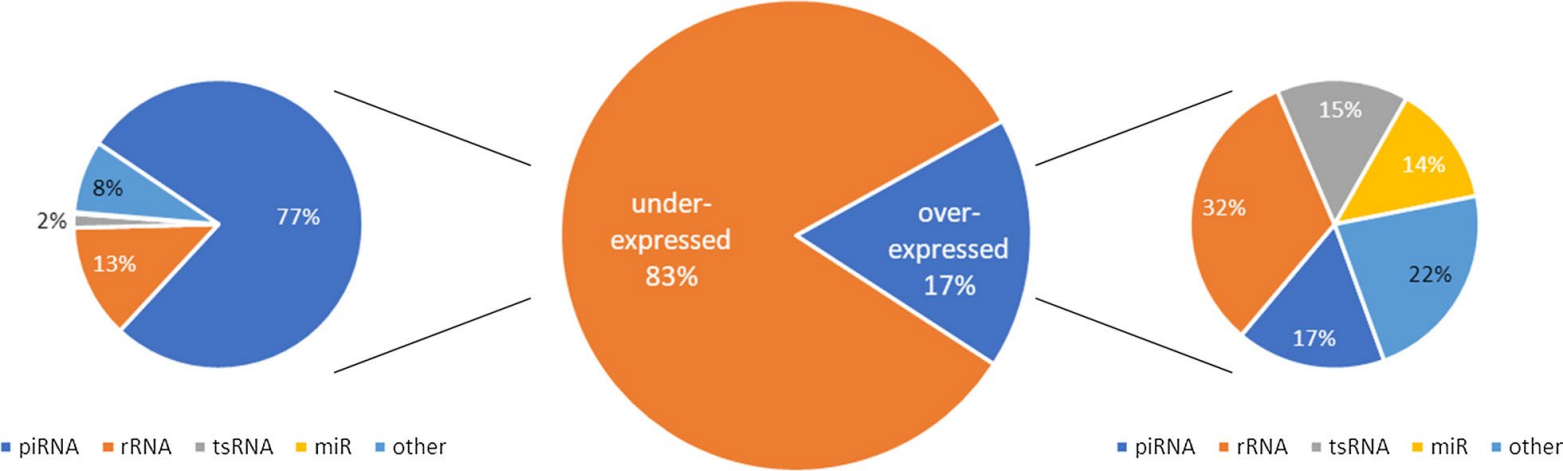
Proportion of sncRNAs families among differentially expressed sncRNA between ejaculated sperm and testis parenchyma (SPZ vs PAR). Differentially expressed sncRNA are mostly under-expressed in SPZ (83%, middle pie chart). Among them 77% are piRNAs, 13% rRNAs and 2% tRNAs (left). Among the over-expressed sncRNAs in SPZ, 32% are rRNAs, 17% piRNAs, 15% tRNAs, and 14% miRNAs (right)

Consistent with the above findings, piRNAs account for the majority of these differentially expressed sncRNAs. For instance, they account for 67% of the 194,062 differentially expressed sncRNAs between PAR and SPZ, while rRNAs, tsRNAs, and miRNAs account for 16%, 4%, and 3%, respectively. As illustrated in Fig. 5, 83% of differentially expressed sncRNAs are under-expressed in SPZ, mainly piRNAs (77%) and rRNAs (13%). Among over-expressed sncRNAs in SPZ, rRNAs, piRNAs, tsRNAs, and miRNAs account for 32%, 17%, 15%, and 14%, respectively.

**Fig. 6:**
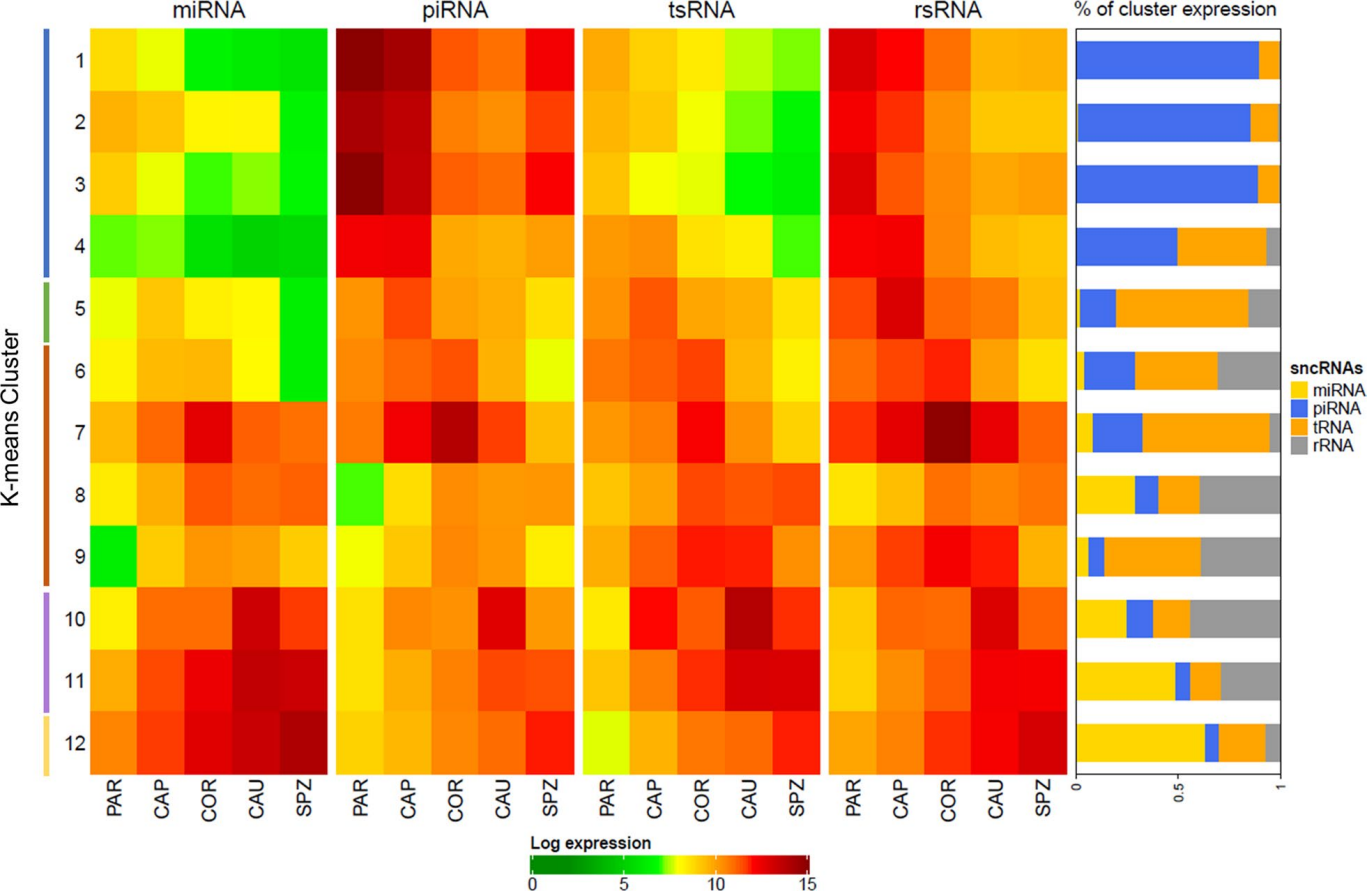
K-means clustering illustrates the diversity of expression profiles among sncRNAs. Total expression (log of normalized expression) of each sncRNA class was plotted as heat maps on the left panel, showing the sncRNA expression from PAR to SPZ (x-axis) per cluster (y-axis). Clusters are grouped according to the region of highest expression: clusters 1–4 gather sncRNAs whose expression peaks at PAR , cluster 5 at CAP , clusters 6–9 at COR , clusters 10–11 at CAU  and clusters 12 at SPZ . The proportion (%) of cluster expression attributable to each sncRNA class is provided as a bar chart on the right panel

K-means clustering was performed with all sncRNA classes to cluster differentially expressed sncRNAs in 12 groups according to their expression profile (Additional file 1: Figure S1) and search for particular expression patterns as well as enrichment in specific biological processes and pathways. As illustrated by the heat map in Fig. 6, a diversity of expression profiles was observed and K-means tended to cluster the expression of specific sncRNA classes (Fig. 6, right panel and Additional file 3: Table S10), in good agreement with the general trends of a decrease in piRNAs and an increase of miRNAs, tsRNAs, and rsRNAs.

For instance, 91% of piRNAs were found in clusters 1–4, which show a global decrease in expression from PAR to SPZ. Of note, the expression of piRNAs belonging to clusters 1–4 was reduced by 98% between PAR and CAU, the major decrease occurring between PAR and CAP (63% due mainly to clusters 1 and 3), while piRNAs were downregulated between CAP and COR (cluster 4 and to a lesser extent cluster 2), accounting for a 35% decrease in expression (Additional file 3: Table S11).

Overall, 95% of miRNAs showed increasing trends, peaking mostly at CAU (clusters 6–9, accounting for 43% of expression) or semen (cluster 12, accounting for 41% of expression). In contrast, 9% of piRNAs showed a more complex profile, with a transient increased expression at either epididymis caput (1% of piRNA and expression), corpus (4% of piRNA, accounting for 11% of expression), cauda (2% of piRNA, accounting for 4% of expression), or ejaculated semen (1.5% of piRNA and expression). Likewise, 3% of miRNAs, together accounting for only 1% of miRNA expression, were found to decrease in expression along the male tract (clusters 1–4). Predominant tsRNA expression was identified at CAU (clusters 10–11) and SPZ (cluster 12) gathering 55% of tsRNA (63% of expression). In contrast, a more balanced distribution was observed for rsRNA, except for clusters peaking at COR (clusters 6–9, accounting for 28% of rRNAs and 50% of expression).

**Fig. 7:**
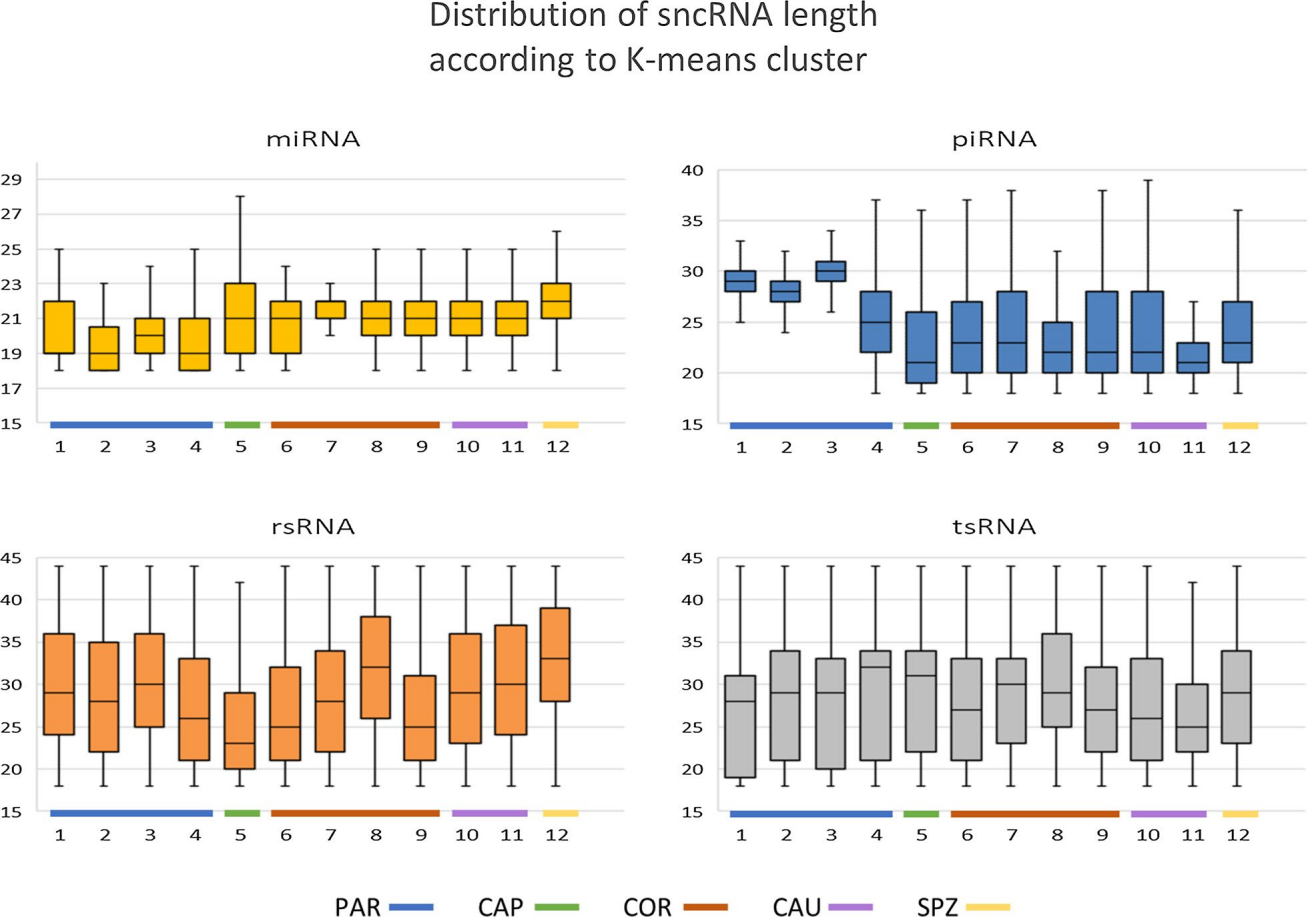
Distribution of read length in each K-means cluster. The distribution of read length in each K-means cluster revealed variations in miRNA and piRNAs lengths among clusters. Indeed, miRNAs appeared to be smaller and piRNAs longer within clusters 1–4 peaking at PAR

Interestingly, while the distribution of sncRNA length within the sncRNA classes was consistent with the expected size for each sncRNA class, specific patterns were observed for some clusters (Fig. 7). For instance, miRNAs appeared to be shorter within testicular clusters (1–4) compared to others. Greater length heterogeneity was also observed according to clusters, indicative of variable intensity of RNA editing mechanisms. However, the most striking variation was observed for piRNAs, whose length appeared to be 30nt at PAR (clusters 1–4), compared to 20-25nt for all other clusters.

**Fig. 8:**
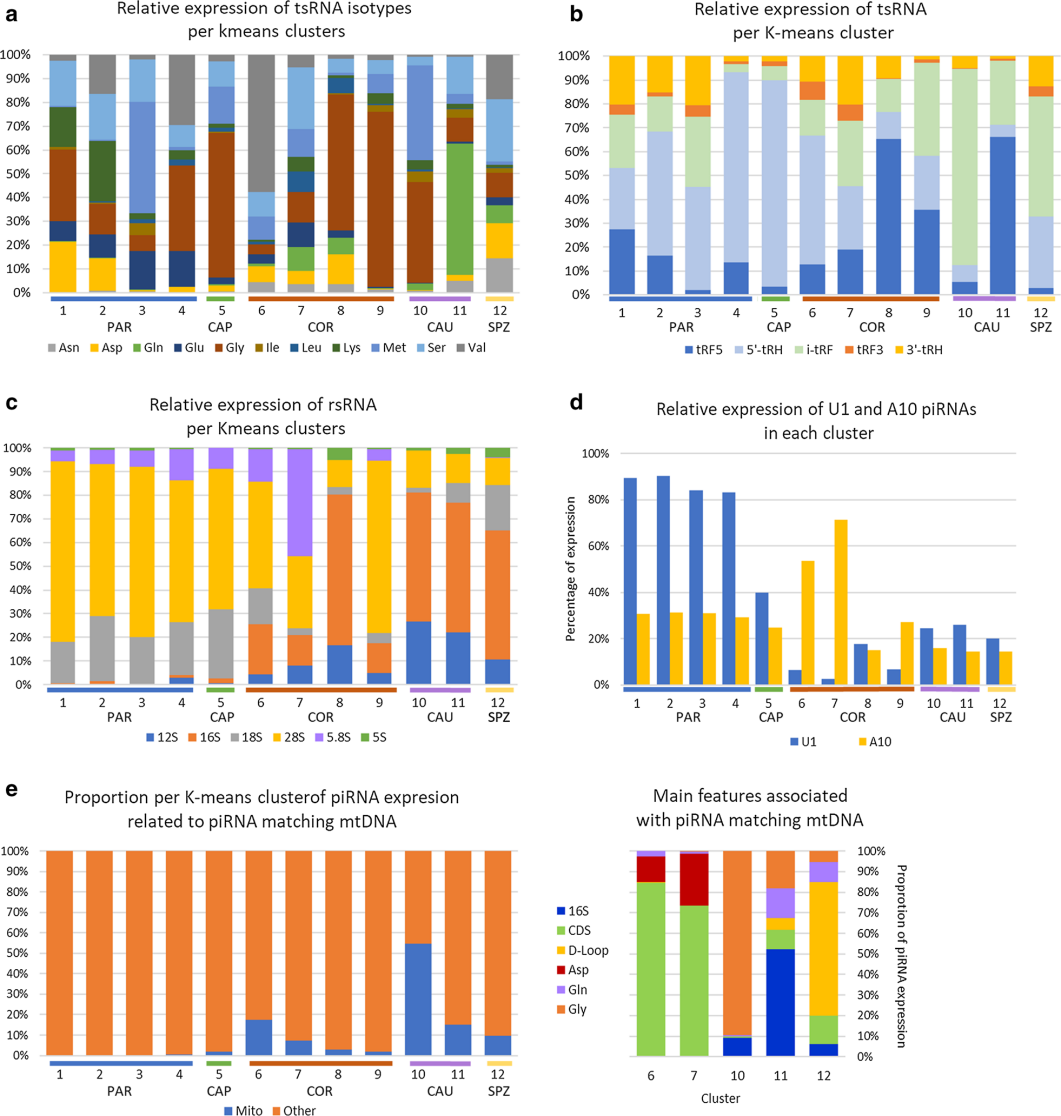
Expression of sncRNA sub-groups per K-means clusters. CAP (caput), COR (corpus), CAU (cauda) epididymis, and ejaculated sperm (SPZ). **a** The proportion of expression attributable to each tsRNA isotype was computed per cluster, showing that some clusters tend to gather the expression of specific sncRNA sub-groups. For instance, Glycine isoacceptors account for a large proportion of expression within clusters 5, 8, and 9 (58%, 56%, and 70%, respectively), while glutamine and valine isoacceptors account for about 54% of expression within cluster 11 and 6, respectively. **b** Regarding tsRNAs, 5'-tRHs account for a large proportion of expression within clusters 2, 4, 5, and 6 (52%, 80%, 86%, and 54%, respectively), while tRF5s account for 66% of clusters 8 and 11 expression. Likewise, i-tRFs account for 50% and 82% of expression within clusters 10 and 12, respectively. **c** Specific patterns were also observed for rRNAs. For instance, 16S account for most of the expression within cluster 8 (64%) as well as 10–12 (55%), while 28S accounts for more than 70% of expression within clusters 1, 3, and 9. **d** A diversity of nucleotide composition was also observed according to K-means clusters. U1 piRNAs account for more than 80% of expression within clusters 1–4, peaking at PAR. In contrast, expression within clusters peaking at COR, CAU, and SPZ was dominated by non-U1 piRNAs. No gross enrichment for an A residue was observed at position 10, except for clusters 6 and 7 peaking at COR. **e** piRNAs perfectly matching mtDNA account for 18% and 55% of piRNA expression within cluster 6 and cluster 10, respectively. Mitochondrial features associated with these piRNAs were mainly coding sequences (CDS, clusters 6 and 7), 16S rRNA (cluster 11), and tRNA-Gly (cluster 12)

In addition, K-means tended to cluster the expression of some specific sncRNA sub-groups (Fig. 8 and Additional file 3: Tables S6–S8). For instance (Fig. 8a), Glycine isoacceptors account for 58%, 56%, and 70% of expression within clusters 5, 8, and 9, respectively, but Glycine isoacceptors are mostly expressed in clusters 9 and 10 (22% and 43%, respectively). Glutamine isoacceptors account for 54% of cluster 11 expression, and conversely, this cluster accounts for 85% of Glutamine isoacceptor expression. Specific patterns were likewise observed for tRFs (Fig. 8b) and rRNAs (Fig. 8c).

A diversity of nucleotide composition was also observed according to K-means clusters, suggesting changes in the piRNA biogenesis along the male reproductive tract (Fig. 8d). Indeed, clusters 1–4, whose expression peaks at PAR, were enriched (> 80%) in U1 piRNAs, while other clusters were dominated by non-U1 piRNAs. No enrichment for A10 piRNAs expression could be observed, except for clusters 6 and 7 peaking at COR, where the proportion of expression attributable to A10 piRNAs reached up to 70%.

Interestingly, when studying the frequency of U1 and A10 piRNAs according to length and K-means clusters (Additional file 3: Table S12, left panel), piRNAs gained at CAP (Cluster 5) showed a shorter length compared to PAR piRNAs as well as no U1 and no A10 bias. Among the 1158 small piRNA (≤ 24nt) from Cluster 5, 86% were found to align perfectly on larger PAR piRNAs from clusters 1–4 (Additional file 3: Table S12, right panel). Furthermore, a bimodal length distribution was observed at CAU (peak at 22nt and 31nt) where clusters 10–11 gather short piRNA having a slight U1 bias and A10 enrichment, as well as long piRNAs having a slight U1 bias (40%) but no A10 signature. A multimodal length distribution was observed at SPZ (cluster 12), with at least three piRNA populations having distinct features: 22nt piRNAs with slight U1 bias and A10 enrichment, 24nt piRNAs with no U1 bias but A10 signature, and 29nt piRNAs with U1 bias and no A10 enrichment. These changes thus suggest that despite similar expression trends, U1 and A10 piRNAs belonging to the same cluster may be subjected to differential regulation.

Sperm piRNA sequences were mapped to bovine mitochondrial genome in an attempt to further characterize this sncRNA class. A total of 19,872 piRNAs were identified, which perfectly matched mtDNA. These piRNAs were mainly gathered in a few K-means clusters peaking at COR, CAU, and SPZ, accounting for 18%, 8%, 55%, and 15% of piRNA expression within cluster 6, 7, 10, and 11, respectively. Mitochondrial features associated with these piRNAs were mainly coding sequences in clusters 6 and 7, 16S rRNA in cluster 11, and tRNA-Gly in cluster 12. In addition, based on the piRBase biogenesis classification, piRNAs were found to primarily derive from intergenic (80%), genic (6%), and LINE-rich (6%) regions, with slight variations according to clusters (Additional file 3: Table S5).

### Biological processes and pathways associated with differentially expressed sncRNAs

A search for biological processes and molecular pathways associated with miRNA targets was performed for each K-means cluster, highlighting several enriched GO terms and Kegg pathways (Additional file 2: Figure S2 and Additional file 3: Tables S13–S15). For instance, clusters 1–4 were shown to gather miRNAs targeting genes involved in cell cycle and its regulation (G1/S transition, cell cycle checkpoint), as well as organelle or compound degradation (ubiquitination and ERAD pathways) and response to stress. Functional annotation of genes targeted by clusters 5–11, whose expression peaks at epididymis, showed enrichment in protein metabolism-related terms, regulation of signaling (signal transduction, peptide secretion), response to stress (regulation of intrinsic apoptotic signaling pathway), cell differentiation, heterochromatin assembly, and embryo development. Genes targeted by cluster 12, with an expression increase in ejaculated sperm, were found to be involved in cellular and organism development (mesenchyme migration, skeletal morphogenesis, angiogenesis, and embryonic morphogenesis), proliferation, and migration and regulation of transcription.

## Discussion

Sperm RNAs have long been considered as spermatogenic leftovers with no further function [28, 29], but a more dynamic picture has emerged recently and the sperm sncRNA profile has been shown to be dynamically modified as the cells migrate through the epididymis [20], possibly through direct transfer of sncRNA from epididymosomes to spermatozoa [13]. Our study was thus designed to study the dynamics of cattle sperm sncRNAs from spermatogenesis to final maturation.

A sufficient number of spermatozoa could be collected, without somatic cell contamination, enabling the preparation and sequencing of good quality RNAs using our previously described protocol. As a result, comprehensive repertoires of cattle sperm sncRNAs could be established at five maturation stages along the male tract. One caveat of our study is related to the fact that the sequencing “real estate” available for a given group of sncRNAs within a sample is affected by the quantity and overall expression of the other sncRNAs in the sample. To limit the potential associated bias and enable a comparison of the relative expression between regions, we performed a between-sample normalization using the relative log expression approach in the DESeq2 package. However, this strategy precludes any conclusions about absolute expression levels. For instance, it is difficult to evaluate whether the increase in miRNA expression in epididymis results from increased expression of this sncRNA class or a decrease in piRNA expression. Despite this limitation, our work provides a detailed picture of sperm sncRNA spatiotemporal dynamics.

### The primary cattle sncRNA sperm content is dramatically remodeled as sperm mature along the epididymis

The sperm sncRNAome showed great plasticity and very large differences in terms of sperm sncRNA content were observed, making it possible to discriminate between sperm samples collected at various maturation stages along the male tract.

One of the most striking findings of our study is the observed enrichment of piRNAs within testis parenchyma relative to the sperm fraction isolated from more distal (corpus and cauda) regions of the epididymis as well as ejaculated sperm. Conversely, an increase in miRNA and tRNA content was observed from testis parenchyma to ejaculated sperm, while rRNAs showed a particular expression profile, peaking at epididymis corpus. Overall, our data are consistent with previous studies in mouse and recapitulate prior findings, both in terms of sncRNA content and global expression trends [12, 19, 30–33]. However, data unavailability as well as species and/or methodological differences precludes a detailed comparison with these previous findings. For instance, Sharma et al. [30] identified tRFs as the primary small RNA population in epididymal sperm but read mappings to rRNAs were discarded before normalization. Here, rRNAs were kept in the analysis and were identified as the major sncRNA population in epididymis corpus. We also report an increase in tRFs expression in epididymis. In addition, among 1.6 million sncRNA sequences described by Chu et al. in mice, only 56,316 perfectly match our bovine sequences (71% rRFs, 15% piRNA, 9% tRFs, and 5% miRNAs). The remaining sequences may reflect both species specificity and the plethora of isoforms produced through RNA editing mechanisms. In this respect, concordance between mouse and bovine increases when considering canonical miRNAs instead of isomiRs represented by individual reads. For instance, about 45% of bovine sperm miRNAs were also described in mouse, including several key miRNAs involved in cell differentiation, proliferation, spermatogenesis, or embryo development (miR-10, miR-29, miR-34, miR-100, miR-148, and miR-191) [19, 31].

Beyond the bulk differences in abundance of broad classes of small RNAs, K-means clustering was performed to cluster sncRNAs based on their spatiotemporal expression profiles, highlighting specific patterns of expression for individual sncRNAs and confirming that the sperm sncRNAome does not simply reflect a legacy of spermatogenesis. Indeed, four different expression profiles were observed for sncRNAs downregulated from PAR to SPZ, differing by the timing and intensity of expression decrease, ranging from 76 to 99% according to sncRNA classes and clusters. Likewise, four profiles were associated with increased expression at epididymis corpus, two at epididymis cauda, and one in ejaculated sperm. Altogether, our data suggest at least three major waves of drastic changes in sperm sncRNA content, first between testis and epididymis corpus, then at epididymis cauda and later in ejaculated sperm. Indeed, the transition from testis parenchyma to epididymis corpus is characterized by a shift from piRNAs to other sncRNA classes, following a drastic decrease in expression of sncRNAs belonging to clusters 1–4, together with a slight increase in miRNA and tsRNA expression (clusters 2–11) and a transient burst in rsRNA expression at COR (cluster 6–9). The second wave of change between epididymis corpus and cauda is characterized by a decrease in rsRNA expression associated with an increase in miRNA and tsRNA expression. The third wave in ejaculated sperm is characterized by an increase in miRNA and a decrease in tsRNA expression.

Encapsulation within the cytoplasmic droplet and ejection upon epididymal entry has been proposed as a likely mechanism to explain the clearance of testicular piRNAs [30]. In cattle, observations on freshly fixed luminal contents suggested that the loss of cytoplasmic droplet occurs at the time of ejaculation [34], making this hypothesis unlikely in vivo. However, in this study, no cytoplasmic droplet could be observed at epididymis cauda, suggesting a loss between epididymis caput and corpus, whatever the reason (e.g., shearing forces due to centrifuge steps). Assuming that cytoplasmic droplets contain no sncRNAs or that their content is similar to the remaining sperm head cytoplasm, their ejection will result in a drastic increase or, respectively, decrease in expression of all sncRNA classes, without any change in their relative proportion. Such an event cannot thus be detected in our NGS study, due to the between-sample normalization procedure. Alternatively, if cytoplasmic droplets are enriched in specific sncRNAs, ejection will result in relative proportion changes that will be observed in our dataset. Since sncRNAs downregulated from PAR to SPZ were clustered according to four different expression profiles of variable intensity of expression decrease, the second option seems more likely. In addition, while expression of sncRNA belonging to clusters 2 and 4 decreases from epididymis caput to corpus, a greater drop in expression was observed between parenchyma and epididymis caput for piRNA belonging to cluster 1 and 3. This suggests that cytoplasmic droplet ejection alone cannot account for the observed changes and other mechanisms may also be at work to account for these piRNAs belonging to clusters 1 and 3.

Interestingly, the epididymal epithelium was shown to produce epididymosomes secreted into the intraluminal compartment, mainly at the initial segment, followed by the cauda, caput, and corpus regions [35]. Interestingly, studies have provided strong evidence that epididymosomes from different segments vary in size [36], interact differently with maturing spermatozoa [37] and have a different sncRNA content [13]. Thus, it has been proposed that epididymosomes (and maybe other extracellular vesicles) may serve as cargo to supplement maturing spermatozoa with sncRNAs during transit through the epididymis, notably miRNAs and tRFs [13, 30, 38]. In particular, substantial variations were observed in epididymosome-borne miRNAs and tsRNAs profiles along the epididymis, with variation in proportion of sncRNA class and relative abundance of each sncRNA as well as increase in profile complexity between the proximal and distal epididymal segments [13]. In good agreement with an external supply of sncRNAs, our findings in cattle mirrors changes described in mouse epididymosomes. Indeed, we observed a rise in miRNAs and tsRNAs relative expression along epididymis, as well as an increase in profile complexity, with about 20% and 10% more miRNAs and tsRNAs in cauda relative to caput sperm.

Moreover, a few percent of piRNAs were identified, which escape the general decreasing trend and peak at epididymis or ejaculated sperm. Interestingly, particular length distributions were observed for these piRNAs, which were shorter than parenchyma piRNAs and showed a distinct nucleotide composition, with mostly non-U1 piRNA, and a strong A10 signature for sncRNA peaking at COR (clusters 6–9). Of note, somatic piRNAs and piRNA-like exhibit a diversity of properties according to tissues and have been described at least as short (18–24 nt) piRNAs with no specific features, mid-size (24–27 nt) piRNAs exhibiting U1 bias (80–90%) and A10 enrichment (40–50%) [39–42] or long (28–32 nt) piRNAs with U1 bias (50–80%) with a distinct signature associated with a yet to be identified biogenesis pathway [43]. In particular, piRNAs peaking at COR gather abundant short piRNA resembling described somatic piRNA and 30nt piRNAs having a ping-pong signature. It is thus tempting to speculate that piRNAs gained at epididymis or later may also originate from interactions with epididymosomes or other extracellular vesicles. However, epididymosomes seem to harbor only small amounts of piRNA [44] and co-incubation of epididymosomes and spermatozoa in vitro does not result in substantial piRNA transfer [12]. In addition, piRNAs gained at epididymis caput (Cluster 5) do not feature typical characteristics of both somatic and pachytene piRNAs. Indeed, they show a particular size distribution, with no peak and exhibit an overall shorter length and a less pronounced U1 bias compared to PAR piRNAs, with no A10 signature. Interestingly, 86% of small piRNA (≤ 24nt) from Cluster 5 were found to align perfectly on larger piRNA (≥ 25nt) from Clusters 1–4, which gather most piRNAs produced at testis parenchyma. This finding suggests that these small piRNAs may feature degradation products or isopiRs produced by editing during the transit from parenchyma to epididymis caput. Alternatively, they may only reflect false positives present in primary databases [45]. It has also been proposed that piRNA gained at epididymis may be produced in situ by sperm following a particular unknown biogenesis pathway [44]. Given that epididymal sperm nuclear gene expression is completely repressed, this latter hypothesis seems unlikely. However, piRNAs may be produced by the mitochondrial genome [46] and may be derived from rsRNAs or tsRNAs [47]. Thus, it could be assumed that in situ production may rely on PIWI processing of rsRNAs or tsRNAs, eventually expressed by mitochondria, and/or mitochondrial expression of piRNAs, which could explain the lack of typical features observed for some piRNAs. In good agreement with this hypothesis, about 8% and 40% of piRNA expression at epididymis corpus and cauda, respectively, were found to be associated with piRNA matching to mtDNA, mainly coding sequences, 16S rRNA and tRNA-Gly. Further studies, including more detailed bioinformatics analysis, as well as NGS sequencing of bovine caput, corpus, and cauda epididymosomes, will be required to further explore these hypotheses.

Of note, although some authors argue that PIWI proteins and piRNAs are depleted in late spermatogenesis stages [48], our findings agree with others reporting that piRNAs exist in ejaculated spermatozoa, opening up the possibility of their role during maturation, fertilization, or embryonic genome activation [49, 50].

A search for sncRNA molecular function, including in silico search for miRNA targets and GO enrichment analysis, was performed for each K-means cluster to provide insights into their putative biological role at each maturation stage.

### Sperm sncRNA, a vestige of spermatogenesis

Downregulated clusters 1–4 show a progressive decrease in expression of sncRNAs from testicular parenchyma to other regions and may thus gather sncRNAs remnant from spermatogenesis. These clusters are enriched in piRNAs resembling pachytene piRNAs (80% U1 piRNA located in intergenic regions), which are known to be crucial for the global mRNA decay in spermatocytes and spermatids as well as the massive turnover of mRNAs in late spermiogenesis [51, 52]. Enriched GO terms and Kegg pathways associated with predicted miRNA targets highlight relevant biological processes for spermatogenesis, such as cell proliferation (regulation of cell cycle and G1/S transition) as well as protein ubiquitination and response to stress. For instance, hyperthermia or oxidative stress has been shown to trigger ER stress and impair spermatogenesis [53, 54], illustrating the key role of ER stress and UPR signaling cascades in spermatogenesis. Likewise, the ubiquitin–proteasome pathway plays a key role at several stages of spermatogenesis (meiosis, histone–protamine transition, acrosome biogenesis, and spermatozoa maturation) to remove many proteins and organelles and facilitate the formation of condensed sperm. Here, UBE2D3, UBE2I, and UBE2J1 were identified as targets of cluster 4 miRNAs. Knock-out mice lacking ubiquitylation enzymes such as UBE2J1 suffer from male sterility associated with flagella and acrosomal defects [55]. Interestingly, piRNAs have been shown to induce the ubiquitination and degradation of MIWI through the APC/C proteasome pathway [48], a process essential for maturation from late spermatid to sperm.

### Epididymal sncRNA modifications: a role for sperm maturation?

Since spermatozoa are considered to be transcriptionally and translationally silent, a role in sperm maturation seems to be unlikely. However, two studies have proven that contrary to the accepted dogma, mRNA are translated by mitochondrial-type ribosomes during capacitation and new proteins are thus synthesized as part of the capacitation process or to replace degraded proteins [56, 57]. Moreover, active transcription and translation have been observed in sperm mitochondria and proved to be essential for mitochondrial activity and regulation of high-speed linear motility through ATP level in low glucose condition [58]. In this respect, assuming that such mechanisms are also operational during maturation, sperm sncRNAs may have a role in regulating energy metabolism, in line with the acquisition of progressive motility by spermatozoa during epididymal transit. For instance, the autophagy pathway (Kegg bta04136) was found to be targeted by miRNAs whose expression peaks at epididymis. This pathway has been associated with regulation of mitochondrial function [59] and human sperm motility [60, 61]. In addition, spermatozoa are highly susceptible to oxidative stress [62]. In this respect, it is worth mentioning that enriched GO terms include regulation of response to stress and tsRNAs have been proposed to be involved in the oxidative stress response [63].

### Enrichment in specific sncRNAs of ejaculated sperm: a role in embryo development?

Sperm RNAs can be delivered to the oocyte during fertilization and remain stable until the onset of embryonic genome activation [64]. Treatment of mature sperm to remove sperm-carried RNAs results in a decrease of blastocyst formation rate [65], and some studies suggest that sperm RNA may act as an additional source of paternal hereditary information, which may be essential for fertilization and/or influence early embryo development [66] and the long-term phenotype of the offspring [67, 68]. Recently, sperm sncRNAs gained at epididymis cauda were shown to be crucial for embryonic development after the blastocyst stage in mice [23]. Interestingly, among miRNAs belonging to clusters 10–12 peaking at epididymis cauda or ejaculated sperm, some have already been described as involved in embryo devolvement. For instance, bta-miR-100, one highly expressed miRNA in ejaculated sperm, has been proposed as one of the main factors associated with the initiation of pluripotency [69, 70] and bta-miR-191, the second most expressed miRNA in our dataset has been associated with fertilization rate and embryo quality in humans [71]. Likewise, miRNA-34c is known to enhance the germinal phenotype of cells already committed to this lineage during spermatogenesis [6], but is also a key player in murine early embryo development [72–74]. In addition, clusters 10–12 were found to be associated with GO terms related to embryo development such as developmental process, embryonic morphogenesis, as well as cell proliferation, differentiation, and migration. Beside miRNAs, rsRNAs, tsRNAs, and some piRNAs are also gained post-epididymis, which may impact embryo development and be involved in trans-generational epigenetic inheritance. Indeed, increasing evidence suggests that sperm RNA-encoded information is decoded in early embryos to control offspring phenotypes [68], as illustrated by altered metabolic pathways in early embryo and induced metabolic disorders in F1 offspring produced by injection into zygotes of sperm tsRNA (mainly tRF5s) from males subjected to a high-fat diet [75]. Of note, tRF5s and i-tRFs are the most enriched tsRNA subpopulation in clusters 10–12. Likewise, tRNA-Gln-TTG, which is enriched exclusively in cluster 11, have been shown to participate in the early cleavage of porcine preimplantation embryos [76] and injection of tRNA-Gly-GCC fragments into zygotes results in slowdown of embryo development [30].

Altogether, these findings suggest both a distinct role of sncRNAs at each maturation stage and coordinated actions of several sncRNA classes at a given maturation stage. In the context of transcriptional and translational inactivity, several hypothetical mechanisms can be proposed to explain sncRNA effects, including regulation of ribosome biogenesis to adapt the mitochondrial translational machinery, use of alternative pathways to produce in situ sncRNAs (e.g., mt-piRNAs and recycling rsRNAs/tsRNAs to produce piRNAs), followed by chain reactions mediated by the interplay with DNA methylation, histone marks, and transposon elements as well as induced metabolic changes [68].

## Conclusions

Mature sperm carries thousands of sncRNAs, including miRNAs, piRNAs, rsRNAs, and tsRNAs, whose function remains unclear, although growing evidence suggests that they play a role in sperm maturation and are involved in paternal epigenetic inheritance. Here, we confirm that cattle sperm sncRNAs are far from being just remnants of the spermatogenic cycle, in good agreement with previous studies in mouse. Rather, we show that sncRNA profiles are dynamically modified as the sperm transit through the epididymis, due to the combination of multiple factors such as loss of the cytoplasmic droplet, intracellular degradation, and interaction with epididymosomes. In addition, we identified piRNAs whose production is unlikely to be attributed to epididymosomes, suggesting in situ production and/or modification of sncRNAs by sperm, a thrilling finding given that epididymal sperm nuclear gene expression is supposed to be completely repressed. Further studies will be required to decipher the underlying mechanisms. In addition, sncRNA expression profiles were used to provide insight into the putative role of sperm sncRNAs. Our preliminary in silico analysis suggests a role during sperm maturation for epididymal sncRNA and embryo development for sncRNAs expressed in ejaculated sperm. This finding warrants further investigations, which may benefit from these results to identify sncRNAs of interest and design relevant experiments.

## Methods

### Semen collection

Semen was collected from three commercial Holstein bulls (60.3 ± 0.7 month of age) following the standard protocol used by the semen production center (UNION EVOLUTION, France). Briefly, after several quality controls (ejaculate volume, sperm concentration, and global motility), the freshly collected semen is diluted in Optidyl® (IMV Technologies, L’Aigle, France) to reach the final sperm concentration of 100 million/ml. Following an equilibration time of 4 h at 4 °C, the diluted semen is placed inside French straws. The straws were gradually cooled from 4 to − 140 °C in a DigitCool® (IMV Technologies) and then submerged and stored in liquid nitrogen at − 196 °C (“SPZ”). On the same day, bulls were slaughtered to collect the testis and epididymis and isolate sperm from testicular parenchyma (“PAR”) as well as three regions of the epididymis [caput (“CAP”), corpus (“COR”), and cauda (“CAU”)].

To do so, several small pieces of each region were incubated 20 min in a Petri dish filled with 3 ml Phosphate-Buffered Saline solution (PBS). The supernatant was collected and centrifuged at room temperature 5 min at 2400×*g* to recover the cellular pellet, which was washed once with 1 ml water to lysate somatic cells and twice with 1 ml PBS, to eliminate the content of lysed cells potentially due to scissor cuts (2400 g 5 min). A visual quality control on sperm (global structure and absence of contamination by somatic cells) was then assessed by microscopy. The final sperm pellet was stored in nitrogen.

### Total RNA isolation, quality control, and sequencing

RNA extraction was performed as previously described [27]. Briefly, sperm pellets were homogenized in 100 µl of RLT buffer (Qiagen) supplemented with 1 µl beta-mercaptoethanol and incubated 15 min at room temperature. Then, 1 ml of Trizol (Invitrogen) was added and samples were incubated 5 min at room temperature before adding 100 μl of chloroform. After vigorous shaking for 15 s, samples were incubated 2 min at room temperature and centrifuged at 12,000×*g* during 15 min at 4 °C.

The aqueous phase was transferred to a new collection tube and mixed with 100 µl of chloroform by vigorous shaking. After incubation for 2 min at room temperature and centrifugation at 12,000×*g* during 15 min at 4 °C, the aqueous phase was recovered, and nucleic acids were precipitated overnight at − 20 °C using 1 volume of isopropanol supplemented with 25 µl glycogen (Ambion AM9510, 5 mg/ml). After centrifugation at 12,000×*g* during 15 min at 4 °C, pellets were washed with 75% ethanol and dried under vacuum. Dried pellets were then re-suspended in 12 µl of RNase-free water and incubated 1 h at 4 °C before quality control assessment.

As described previously [27], RNA concentration was assayed using the Qubit® fluorometer (Life Technologies), with the RNA HS Assay Kits (Q32852 Life Technologies) according to the manufacturer’s recommendations. Of note, no RNA could be obtained from Optidyl alone, implying limited technical bias due to the extender on the SPZ sncRNA content. In addition, RT-qPCR was used to detect and quantify miR-125-5p which has been shown to be highly expressed in bovine sperm. To exclude any contamination by somatic RNAs, RT-qPCR was performed to assess expression of the Epididymal Secretory Glutathione Peroxidase gene *(GPX5*), which is known to be expressed by epididymis epithelium cells. Primers were designed from the Bovine sequence (UCSC genome browser) using Primer3web (https://primer3.ut.ee). Their efficiency and specificity were evaluated on bovine genomic DNA.

Preparation of the 15 libraries and sequencing were performed by Qiagen, starting from 42 ng total RNA and using the QIAseq miRNA Library Kit® and Unique Molecular Indexes (UMI). UMIs are random oligonucleotide barcodes used to distinguish identical copies arising from distinct molecules from those arising through PCR amplification of the same molecule. UMI deep sequencing was performed on Illumina HISeq2000, targeting 20 M single-reads (75 bp) per sample.

### Bioinformatics and biostatistics

Our previously described bioinformatic pipeline [27] was slightly modified to take UMI technology into account: de-duplication was performed first based on UMI, to remove reads arising through PCR amplification of the same molecule. UMIs and adaptors were trimmed (UMI-tools) and only one exemplar from identical sequences sharing the same UMI was conserved, associated with the mean quality score calculated at each position. The remaining sequences were then filtered to remove reads shorter than 17nt and sequences containing at least one nucleotide having a Phred quality score under 25. Remaining sequences were then stacked by unique sequences to compute total read counts and mapped on the bovine reference genome (ARS-UCD1.2; BosTau9). A workflow mostly based on miRDeep2 [77, 78] was used to identify and quantify known (miRBase v22) as well as predicted miRNAs. Expression levels were computed both at the miRNA and isomiR levels. In parallel, all unique sequences were annotated using several public databases including Ensembl release 94 (http://www.ensembl.org), RFAM release 14 (http://rfam.wustl.edu), cattle ncRNA from ENA (https://www.ebi.ac.uk/ena), cattle tRNA from GtRNAdb (www.gtrnadb.ucsc.edu), human, mouse and cattle piRNAs from ENA, as well as [79, 80]. All workflow scripts are available on GitHub (https://github.com/SmartBioInf/PAQmiR). Subgroups of tsRNA were identified as previously described [27]: sequences aligned to the beginning of 5′ end of the mature tRNA were classified as tRF5, while those aligned to the 3′ end of mature tRNA were classified as tRF3; sequences generated by a cleavage at the anticodon-loop and producing tRNA halves were classified as 5′-tRHs and 3′-tRHs according to their localization on mature tRNA; internal fragments were classified as i-tRFs. Bovine piRBase databases were used to annotate sperm piRNA according to their target type (LINE, SINE, LTR, or Satellite) or target location (intergenic or genic). To locate piRNA sequences in mtDNA, all sperm piRNA sequences were mapped to bovine mitochondrial genome (ENA accession V006541) using blast (options—task “blastn-short”), keeping only perfect matches along the whole piRNA sequence. Targetscan 7.2 (release March 2018) was used to predict miRNAs targets. Functional annotations and enrichment analysis were done using Gorilla [81] (http://cbl-gorilla.cs.technion.ac.il/) and WebGestalt [82] (http://www.webgestalt.org/). GO results were summarized to produce scatterplots and interactive graphs of enriched terms grouped by semantic similarity using Revigo [83].

The DESeq2 R package (R v3.6.0 and DESeq2 package v1.24.0) [84] was used to estimate library size normalization factors using a median-of-ratios (i.e., relative log expression) approach and test for differential sncRNA expression between regions. Statistical significance was evaluated based on Benjamini–Hochberg adjusted *p* values, with a significance threshold of 5%. The proportion of each sncRNA classes was computed per region based on counts (proportion) as well as normalized expression (relative expression). A between-class analysis (BCA) was performed using the ade4 R package (dudi.bca function). K-means clustering (Lloyd algorithm, 100 random starts and 1000 iterations) was performed using the *kmeans()* function in R on standardized data (read counts were averaged across the 3 bulls for each region before centering and scaling values for each row). The elbow method was used to estimate the optimal number of K-means cluster. To do so, K-means clustering was performed for *k* = 2 to *k* = 25 and the amount of information retained after clustering was computed as the ratio of betweenss and totss. Using *k* = 12 was shown to retain more than 90% of information, with no significant improvement in retained information above 12 clusters. Graphs were plotted using the ggplot2 library.

### RT-qPCR validation of differential expression among regions

Expression of three miRNAs (bta-miR-100: AACCCGTAGATCCGAACTTGT, bta-miR-16a: TAGCAGCACGTAAATATTGGCG and bta-chr27-30001: CGCCGGGGCGGGTTCCGGAGG) as well as two bta-miR-191 isomiRs (CAACGGAATCCCAAAAGC and CAACGGAATCCCAAAAGCAG) was assessed by RT-qPCR on 3 testis parenchyma sperm and 3 ejaculated sperm samples to evaluate their differential expression. LNA primers were supplied by Qiagen. Duplicates were assayed in 10-μl/5-ng qPCR reactions following the miRCURY LNA kit protocol, using a StepOnePlus Real-Time PCR System (Applied biosystems). Amplification curves were analyzed using the StepOne software v2.3 to compute Ct values and analyze the melting curve. Relative expression was computed with the qbase^+^ software (Biogazelle).

## Supplementary Information

**Additional file 1: Figure S1.** SncRNA expression profiles according to K-means clusters. K-means clustering illustrates the diversity of expression profiles among sncRNAs. Standardized normalized expression plots were drawn on separate panels for each cluster. Cluster name and the relative expression of each sncRNA class in each K-means cluster are provided on top of each panel. For instance, miRNA and piRNA account for 0.2% and for 89.8% of expression within cluster1, respectively. Mean standardized normalized expression levels are depicted as red lines for each cluster. K-means clusters are grouped according to the region of highest expression: clusters 1–4 gather sncRNA whose expression peaks at PAR blue bar, cluster 5 at CAP green bar, clusters 6–9 at COR brown bar, clusters 10–11 at CAU violet bar and clusters 12 at SPZ yellow bar.

**Additional file 2: Figure S2.** GO biological processes associated with K-means clusters. For each cluster, miRNA targets predicted by Targetscan were retrieved to explore biological processes and pathways. Gorilla was used to identify enriched GO terms and scatterplots were produced using Revigo, showing enriched terms grouped by semantic similarity. Relevant GO terms are shown for miRNA targets of **a)** clusters 1–4 (peaking at PAR and decrease post testis); **b)** clusters 5–11 whose expression peaks at epididymis; **c)** cluster 12, whose expression increase from PAR to SPZ. The size of each dot is proportional to the number of genes related to the given term (log size) and the color depicts the associated p-value.

**Additional file 3: Table S1.** Mapping statistics across the 15 libraries. **Table S2.** The Sperm miRnome. **Table S3.** Sperm rRNAs. **Table S4.** Sperm tRNAs. **Table S5.** Sperm piRNAs. **Table S6.** Dynamics of piRNAs. **Table S7.** Dynamics of tsRNAs. **Table S8.** Dynamics of rsRNAs. **Table S9.** RT-qPCR validation of differential expression observed by NGS for 5 isomiRs between testis parenchyma and total ejaculated sperm. **Table S10.** Proportion and relative expression of sncRNA per k-means clusters. **Table S11.** Contribution of CAP, COR and CAU regions to the overall decline in expression from PAR to CAU for sncRNA belonging to K-means clusters 1–4. **Table S12.** Detailed study of piRNA nucleotide composition and length per group of K-means clusters. **Table S13.** miRNA targets per k-means cluster. **Table S14.** Enriched GO terms per k-means cluster. **Table S15.** Enriched Kegg pathways per K-means cluster.

## Data Availability

Fastq files were made available to the scientific community at the European Nucleotide Archive (ENA), under primary accession number: PRJEB41989.
