# Supplementary figures and images for: Dynamics of cattle sperm sncRNAs during maturation, from testis to ejaculated sperm
Source: Epigenetics Chromatin. 2021 May 24;14:24. doi: 10.1186/s13072-021-00397-5 (PMC8146655; doi:10.1186/s13072-021-00397-5)

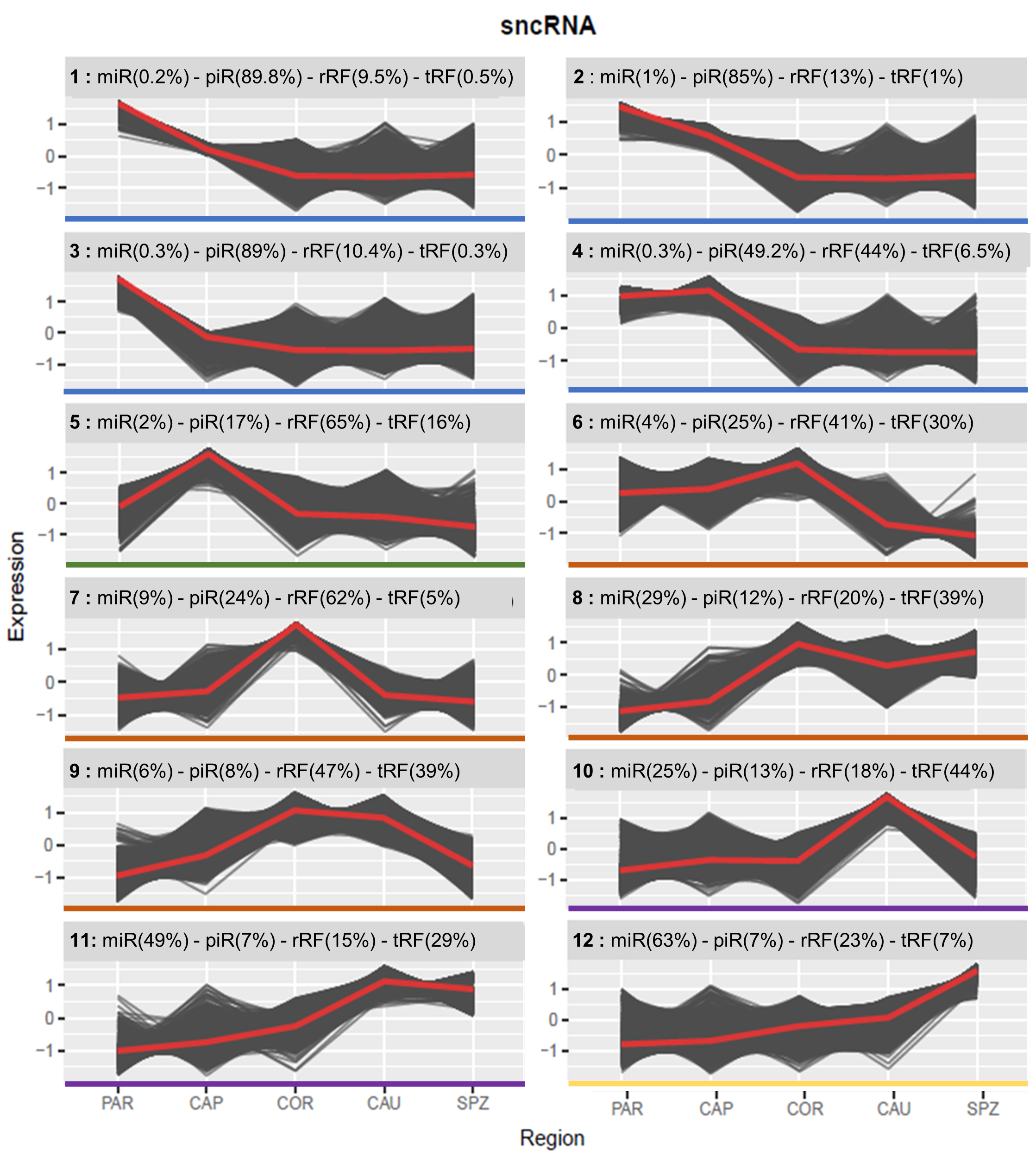

Supplement: Supplementary file 1 — Additional file 1: Figure S1. SncRNA expression profiles according to K-means clusters. K-means clustering illustrates the diversity of expression profiles among sncRNAs. Standardized normalized expression plots were drawn on separate panels for each cluster. Cluster name and the relative expression of each sncRNA class in each K-means cluster are provided on top of each panel. For instance, miRNA and piRNA account for 0.2% and for 89.8% of expression within cluster1, respectively. Mean standardized normalized expression levels are depicted as red lines for each cluster. K-means clusters are grouped according to the region of highest expression: clusters 1–4 gather sncRNA whose expression peaks at PAR blue bar, cluster 5 at CAP green bar, clusters 6–9 at COR brown bar, clusters 10–11 at CAU violet bar and clusters 12 at SPZ yellow bar. [file 13072_2021_397_MOESM1_ESM.tif]

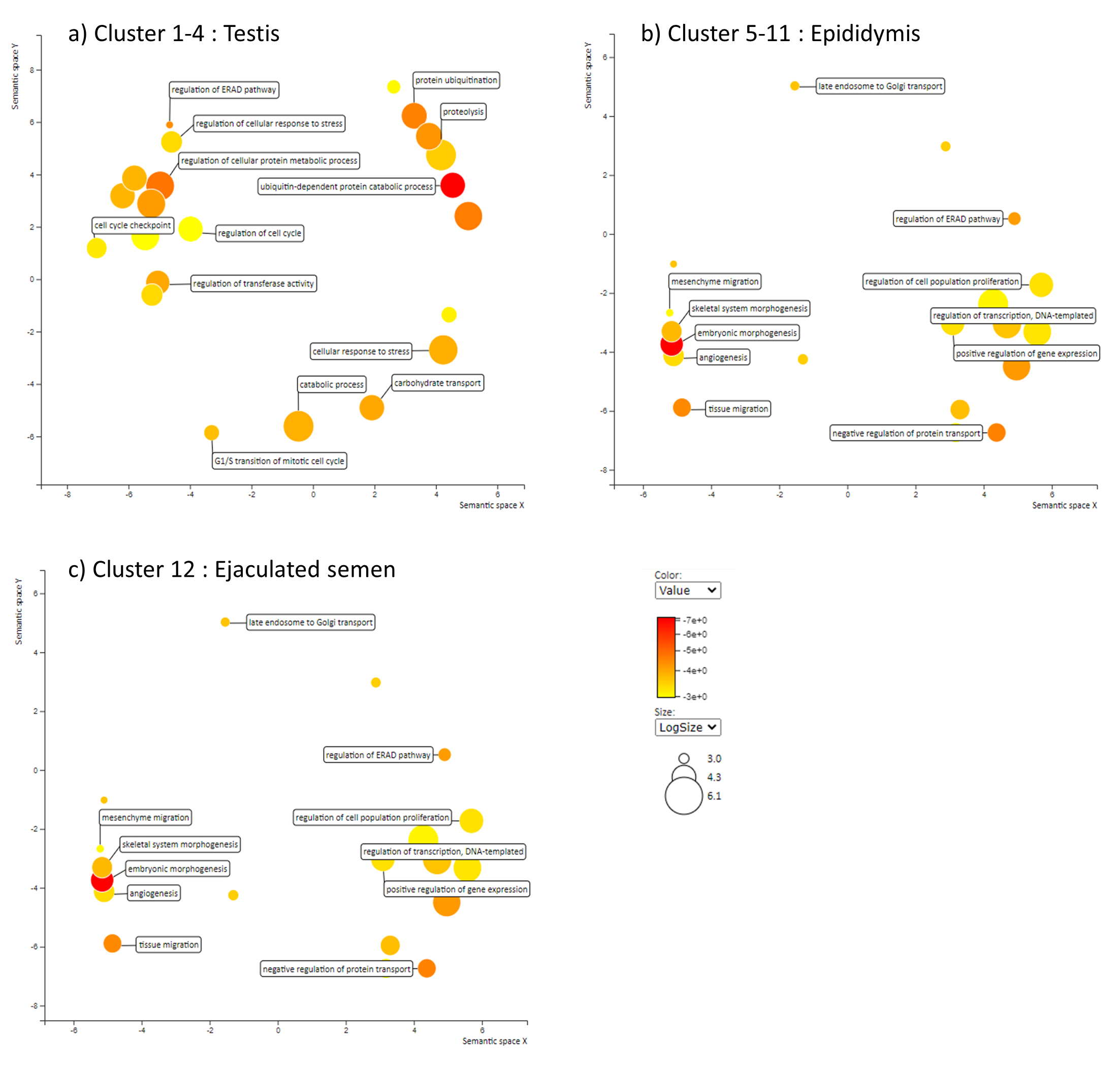

Supplement: Supplementary file 2 — Additional file 2: Figure S2. GO biological processes associated with K-means clusters. For each cluster, miRNA targets predicted by Targetscan were retrieved to explore biological processes and pathways. Gorilla was used to identify enriched GO terms and scatterplots were produced using Revigo, showing enriched terms grouped by semantic similarity. Relevant GO terms are shown for miRNA targets of a) clusters 1–4 (peaking at PAR and decrease post testis); b) clusters 5–11 whose expression peaks at epididymis; c) cluster 12, whose expression increase from PAR to SPZ. The size of each dot is proportional to the number of genes related to the given term (log size) and the color depicts the associated p-value. [file 13072_2021_397_MOESM2_ESM.tif]
